# Industrial hemp biomass negatively affected by herbicide drift from corn and soybean herbicides

**DOI:** 10.1038/s41598-024-78209-5

**Published:** 2024-11-15

**Authors:** Milos Zaric, Bruno Canella Vieira, Barbara Houston, Guilherme Sousa Alves, Sam E. Wortman, Julie Peterson, Greg R. Kruger

**Affiliations:** 1 Department of Agronomy and Horticulture, University of Nebraska-Lincoln, West Central Research, Extension and Education Center, 402 W State Farm Road (W. P. Snyder Building 124), North Platte, NE 69101 USA; 2 Former Department of Agronomy and Horticulture, University of Nebraska-Lincoln, West Central Research, Extension and Education Center, North Platte, NE 69101 USA; 3 Department of Agronomy and Horticulture, University of Nebraska-Lincoln, Lincoln, NE 68503 USA; 4 Department of Entomology, University of Nebraska-Lincoln, West Central Research, Extension and Education Center, North Platte, NE 69101 USA

**Keywords:** Environmental impact, Plant stress responses

## Abstract

The establishment of industrial hemp (*Cannabis sativa* L.) fields near row crops has raised concerns about the potential adverse effects of herbicide drift on hemp production. This study examined hemp susceptibility to drift of herbicides registered for use in corn and/or soybeans. Herbicide solutions (2,4-D, dicamba, glufosinate, glyphosate, imazethapyr, lactofen, mesotrione) were applied separately in the wind tunnel (3.6 m s^−1^ airspeed), simulating drift scenarios, with conventional TP95015EVS (TP) and air inclusion AI95015EVS (AI) flat fan nozzles calibrated to deliver 140 L ha^−1^ carrier volume at 230 kPa. Mylar cards and hemp plants (20–25 cm tall) were placed downwind up to 12 m. Spray deposition from mylar cards was quantified using fluorometry and hemp biomass was collected 21 post application. Results indicated the nozzle design influenced downwind deposition; 5% of spray deposits from the TP nozzle reached 5.9 m downwind versus 2.0 m for the AI nozzle. Glyphosate, glufosinate, and mesotrione caused the highest biomass reductions, with 50% reductions observed at 19.3 (inferred), 8.7, and 9.3 m downwind for TP nozzle, and 4.1, 4.0, and 2.9 m for AI nozzle. These findings suggest herbicide applications at airspeeds of 3.6 m s^−1^ or greater present a risk to nearby hemp fields.

## Introduction

Opportunity for growth and utilization in various industries has led to the adoption of industrial hemp (*Cannabis sativa* L.) in over 30 nations worldwide^1^. Since 2018, industrial hemp can be legally grown for grain, fiber, and pharmaceuticals (i.e., cannabinoids) in at least 48 continental United States (US)^2–5^. Annual hemp product sales for 2018 and 2021 are estimated at nearly $700 and $821 million in the US, respectively^6,7^. As of 2022, Colorado (4087 ha) and Montana (3197 ha) are the two leading states in industrial hemp production, with a significant increase in planted areas since 2018^7^. Specifically, in the North Central growing region of the US, there is expected to be an increase in industrial hemp grown over the next five years, and industrial hemp will be grown in fields adjacent to herbicide-tolerant row crops.

The wide adoption of herbicide-tolerant crops in the US has resulted in an increased overreliance on herbicides for post-emergence (POST) weed management^8–11^. The abundance of POST and broad-spectrum herbicide applications, including glyphosate, glufosinate, dicamba, and 2,4-D, has raised concerns regarding off-target movement^12–15^. Common sources of off-target movement from row crops such as corn and soybean are through herbicide physical or vapor drift that occurs at or after application^13,16,17^. Current research predominantly focused on providing insights into industrial hemp susceptibility to herbicide particle drift (hereafter referred to as herbicide drift), commonly defined as a part of the herbicide application that moves away from the target area by wind-carried droplets^18,19^. Previous studies suggest that about 70% of the sprayed herbicide solution reaches the final target, while the remaining 30% may be lost due to off-target movement and/or runoff^20,21^. Therefore, proper selection of application technology parameters must be considered to improve application efficiency while reducing herbicide drift potential^19^.

Herbicide application is a complex process influenced by many environmental conditions, including wind airspeed, temperature, and relative humidity^22,23^. In addition, sprayer setup, product formulation, tank-mix additives, nozzle type, operational pressure, and boom height can influence herbicide drift potential^24–29^. Of all variables, spray droplet size directly affected by nozzle selection has been considered the most critical variable associated with herbicide drift reduction and subsequent consequences^30^. Some earlier-developed (standard) flat fan nozzles are characterized by a higher proportion of finer droplets than new drift-reducing ones. Although standard and drift-reducing nozzles can have similar performance in terms of effectiveness depending on the product used, nozzles containing a greater fraction of finer droplets (less than 200 µm) are typically more prone to drift^31–34^. Therefore, to minimize herbicide drift, nozzle manufacturers developed drift-reducing nozzle types that generate lower percentages of driftable fines with coarser droplets^22,25,30,35^. One of the differences in drift potential among nozzle types is due to pre-orifice and/or the venturi air-inclusion ports featured in some nozzle designs. In both cases, the aim is to increase droplet size, which will reduce drift potential^25,36,37^ and downwind spray deposition^22,24,38^, as well as consequences to non-target crops.

As an emerging crop, no POST herbicides are registered for industrial hemp in the US^39^. Previous research reported a strong positive correlation between total biomass and hemp fiber (R^2^ = 0.86) and stem biomass yield (R^2^ = 0.97)^40^. Hemp plants exposed to herbicide drift may experience reduced growth and yield, ultimately decreasing total biomass, fiber yield, and stem biomass yield. The strong positive correlation between total biomass and these yield parameters suggests that herbicide drift could have a negative impact on hemp and an overall reduction in economic value. There is limited information on how much industrial hemp biomass production and final yield could be impacted by herbicide drift from adjacent crops^41^. Moreover, if herbicide residues are detected in plant parts used for direct consumption, it could result in severe economic loss or even crop destruction^42,43^. Adverse economic outcomes from off-target pesticide movement have already been reported when high-value, specialty, and food-grade crops are planted near row crops^44–48^. Similar outcomes could be expected for industrial hemp, where consistency in the quantity and quality of harvested materials are critical for either end-industrial use or further processing^2,42,49^.

Previous research on evaluating industrial hemp tolerance to herbicides has been conducted with direct applications over the top of plants using just a single or series of herbicide doses under diverse herbicide application settings^41,50^. As a result, it was determined that most of the evaluated POST herbicide programs, when applied at recommended rates, resulted in detrimental industrial hemp injury and biomass reduction. Ortmeier-Clarke et al.^41^ reported an estimate of the effective dose for 10% industrial hemp biomass reduction for mesotrione (0.005 g ai ha^−1^), imazethapyr (1.5 g ai ha^−1^), lactofen (0.3 to 4.8 g ai ha^−1^), and glufosinate (21 g ai ha^−1^). All earlier studies have evaluated industrial hemp herbicide susceptibility by simulating drift with sub-labeled doses of herbicides applied directly over the top of the plants. However, it is essential to understand the potential impact of herbicide drift applied at recommended labeled rates and drift toward industrial hemp at various distances from the nozzle (i.e., actual simulation of herbicide drift instead of simulated through sub-labeled doses) under high and low herbicide drift potential.

This research aimed to perform droplet size analysis and quantify the impact of herbicide drift on spray deposition and industrial hemp biomass reduction from commonly used herbicides in corn and/or soybean in a low-speed wind tunnel. The hypotheses were that the nozzle type (with distinct drift potentials) and herbicide would influence droplet formation, spray deposition, and industrial hemp biomass reduction.

## Materials and methods

### Study site and plant material

All studies were conducted during the summer and fall of 2020 at the Pesticide Application Technology Laboratory (University of Nebraska-Lincoln, West Central Research, Extension and Education Center in North Platte, NE, USA). A multi-purpose (high-cannabidiol, fiber, and grain) variety of industrial hemp (NWG2730, New West Genetics Inc., Fort Collins, CO, USA) used in this study was obtained as a part of the material transfer agreement (*2020-0369A*) between both institutions. In addition, special permission to grow industrial hemp for research purposes for the 2020 growing season was obtained through the Nebraska Department of Agriculture, granted under the *Industrial Hemp Pilot Research Project* (no special license required).

### Growing conditions

Industrial hemp seeds were planted in 1 L plastic pots containing commercial potting mix (Pro-Mix BX5, Premier Tech Horticulture Ltd, Rivière-du-Loup, Canada). Pots were maintained under greenhouse conditions (30 °C during the day and 20 °C during the night) and irrigated daily with tap water. Plants were fertilized using fertilizer blended with water at 0.2% v v^−1^ (UNL 5-1-4, Wilbur-Ellis Agribusiness, Aurora, CO, USA) as needed. Supplemental lighting was provided using LED lights (520 μmol s^−1^, Philips Lighting, Somerset, NJ, USA) to ensure a 16-h photoperiod and keep plants in a vegetative stage.

### Droplet size measurements

Spray droplet size was quantified in a low-speed wind tunnel with all herbicide solutions (2,4-D, dicamba, glufosinate, glyphosate, imazethapyr, lactofen, and mesotrione) separately sprayed (Table 1) using the highest recommended field labeled rates at 140 L ha^−1^ and two nozzle types including TP95015EVS and AI95015EVS (TeeJet Technologies Spraying Systems Co., Glendale Heights, IL, USA) at 230 kPa. The droplet size analysis for each treatment combination evaluated for this study was measured three times using a laser diffraction instrument in a wind tunnel with a constant airspeed of 6.7 m s^−1^. Each replication consisted of a complete spray plume passing through the measurement area. More information about procedures and wind tunnel setup, operation, study methods, and concept clarifications are described by Creech et al.^51^ and Vieira et al.^52^. Recorded values included D_V0.1_, D_V0.5_, and D_V0.9_ (droplet diameters such that 10, 50, and 90% of the total spray volume is in droplets of lesser diameter, respectively). The percentage of driftable fines, defined as droplet diameters 200 µm or less, was reported as a proportion of the total spray volume. The spray classifications were based on curves from reference nozzles spraying water alone per ASABE S572.3 standard^53^.

**Table 1:** Herbicide solution, Trade name, Weed Science Society of America (WSSA) Mode of Action group, and application rate for solutions evaluated in the spray drift study^a^.

Herbicide solution	Trade name	WSSA Group^b^	Application Rate^c^	Manufacturer
			g ai/ae ha^−1^	
2,4-D	Enlist One®	4	1065	Corteva AgriSciences
Dicamba	Xtendimax®	4	560	Bayer CropScience
Glufosinate	Liberty® 280 SL	12	645	BASF Corporation
Glyphosate	Roundup WeatherMAX®	9	1260	Bayer CropScience
Imazethapyr	Pursuit®	2	70	BASF Corporation
Lactofen	Cobra®	14	220	Valent U.S.A. Corporation
Mesotrione	Callisto®	27	105	Syngenta Crop Protection

^a^A fluorescent tracer 1,3,6,8-pyrene tetra sulfonic acid tetra sodium salt (PTSA) was added at 3 g L^−1^.

^b^Herbicide mode of action as listed by Weed Science Society of America.

^c^Application rate shown as grams of active ingredient (ai) or acid equivalent (ae) per hectare.

### Wind tunnel herbicide drift study

Simulated herbicide drift under controlled conditions was conducted to understand the impact of nozzle selection and herbicide solutions on spray deposition and industrial hemp biomass reduction. Integration of simulated drift in a low-speed wind tunnel as a study method has been determined to nearly generate data observed under the field conditions when similar nozzles were used^52^. Spray deposition and industrial hemp herbicide drift studies were conducted in a complete randomized design with four replications. The study was conducted twice (summer and fall), resulting in two experimental runs. Industrial hemp was used as a bioindicator plant to assess herbicide drift implications on biomass reduction in a wind tunnel following a similar approach reported in previous studies^38,54^.

**Fig. 1:**
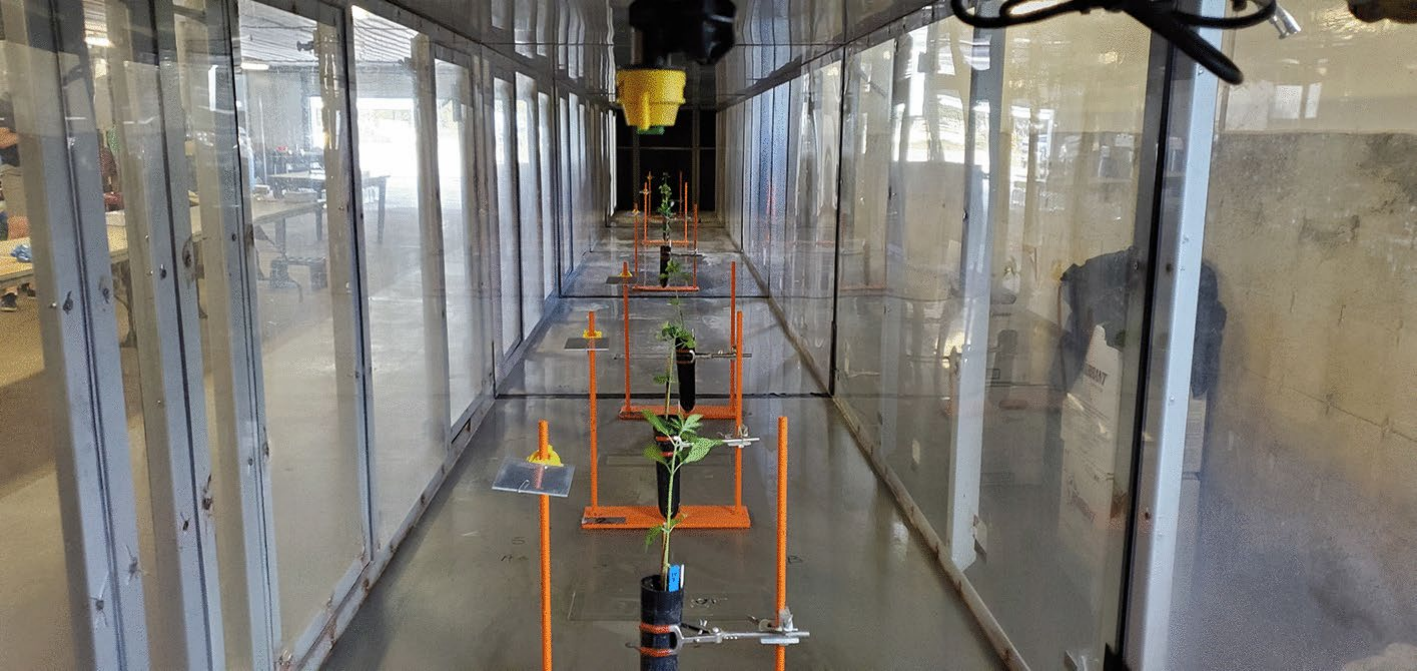
Interior of low-speed wind tunnel with spray deposition collectors and industrial hemp plants positioned at downwind distances from the nozzle.

The seven herbicide solutions (2,4-D, dicamba, glufosinate, glyphosate, imazethapyr, lactofen, and mesotrione) were prepared as specified in Table 1 with the addition of 1,3,6,8-pyrene tetra sulfonic acid tetrasodium salt (PTSA) as a fluorescent tracer (Spectra Colors Corporation, Kearny, NJ, USA) at a concentration of 3 g L^−1^. Herbicide solutions were sprayed at 140 L ha^−1^ and 230 kPa using TP95015EVS (TP) and AI95015EVS (AI) nozzle types under a 3.6 m s^−1^ airspeed in a wind tunnel. The selection of TP and AI nozzle types allowed the evaluation of industrial hemp response to two distinct herbicide drift scenarios with high and relatively low-risk potential, respectively. Spray deposition collectors and hemp plants were placed at downwind distances of 1, 2, 3, 6, 9, and 12 m from the spray nozzle (Fig. 1). Mylar cards (10 cm by 10 cm) (Grafix Plastics, Cleveland, OH, USA) were used as spray deposition collectors. Industrial hemp plants 20–25 cm tall (two or three pairs of true leaves) were 51 cm underneath the nozzle. Each replication encompassed a uniform two-second duration of continuous application, regulated by a digital auto shut-off timer switch (Intermatic Inc., EI 400C, Spring Grove, IL, USA). Mylar cards and plants were removed from the wind tunnel two minutes after herbicide application. The average air temperature and relative humidity during this study were 22–25 °C and 45–50%, respectively.

### Mylar card processing and plant maintenance

To avoid photodegradation of PTSA after application, mylar cards were kept separate in individual pre-labeled bags under dark conditions. Spray deposition for each herbicide solution was quantified by fluorometric analysis. Mylar cards were washed in 40 mL of 10% alcohol solution (91% isopropyl alcohol, PL Developments, Clinton, SC, USA) and prepared with distilled water. After the cards were washed, a 1.5 mL aliquot was removed from each sample bag to fill a glass cuvette. The cuvette was placed inside a fluorimeter (Turner Designs, Trilogy, Sunnyvale, CA, USA) equipped with a PTSA module that uses ultraviolet light to obtain relative fluorescence data (RFU). RFU was converted into ng cm^−2^ to obtain spray deposition percentage and compared to the theoretical application rate of 140 L ha^−1^. Alves et al.^22^ and Vieira et al.^38^ describe additional information about conversion procedures.

After application, hemp plants were maintained in the greenhouse under previously described growing conditions. Plant aboveground biomass was harvested 21 days after application. Biomass was dried in an air-forced dryer at 65 °C to reach a constant weight. Dry biomass weights were recorded and converted into percentage of biomass reduction using Eq. (1):

1 $$br = \left[ {\left( {nt - t} \right)/nt} \right] * 100$$

where *br* represents biomass reduction (%), *nt* is dry biomass (g) of non-treated plants, and *t* is dry biomass (g) of plants exposed to herbicide drift.

### Statistical analysis

The droplet size dataset was subjected to analysis of variance using a generalized linear mixed model (PROC GLIMMIX) in SAS (Statistical Analysis Software, v9.4, Cary, NC, USA). All comparisons were performed within and across nozzle types at α = 0.05 significance using a *Tukey’s* Least Significant Difference test.

For herbicide spray deposition and industrial hemp biomass reduction, the model selection function *mselect* tool in R software (R Foundation for Statistical Computing, Vienna, Austria) was used to compare several non-linear model candidates to fit the data^41,55^. The four-parameter log-logistic function was selected as the best-fit model based on Akaike’s information criterion (data not shown), which was analyzed using the *drc* package in R software following Eq. (2):

2 $$y = c + \{ {d - c/1 + \exp [ {b( {\log x - \log e} )} ]} \}$$

where *y* represents spray deposition or biomass reduction (%), *b* is the slope at the inflection point, *c* is the lower limit of the model (fixed to 0%), *d* is the upper limit (fixed to 100%), and *e* is the inflection point (distance to 50% spray deposition (m) or biomass reduction (%))^56,57^. Data from the two experimental runs were combined.

## Results and discussion

### Droplet size distribution

**Fig. 2:**
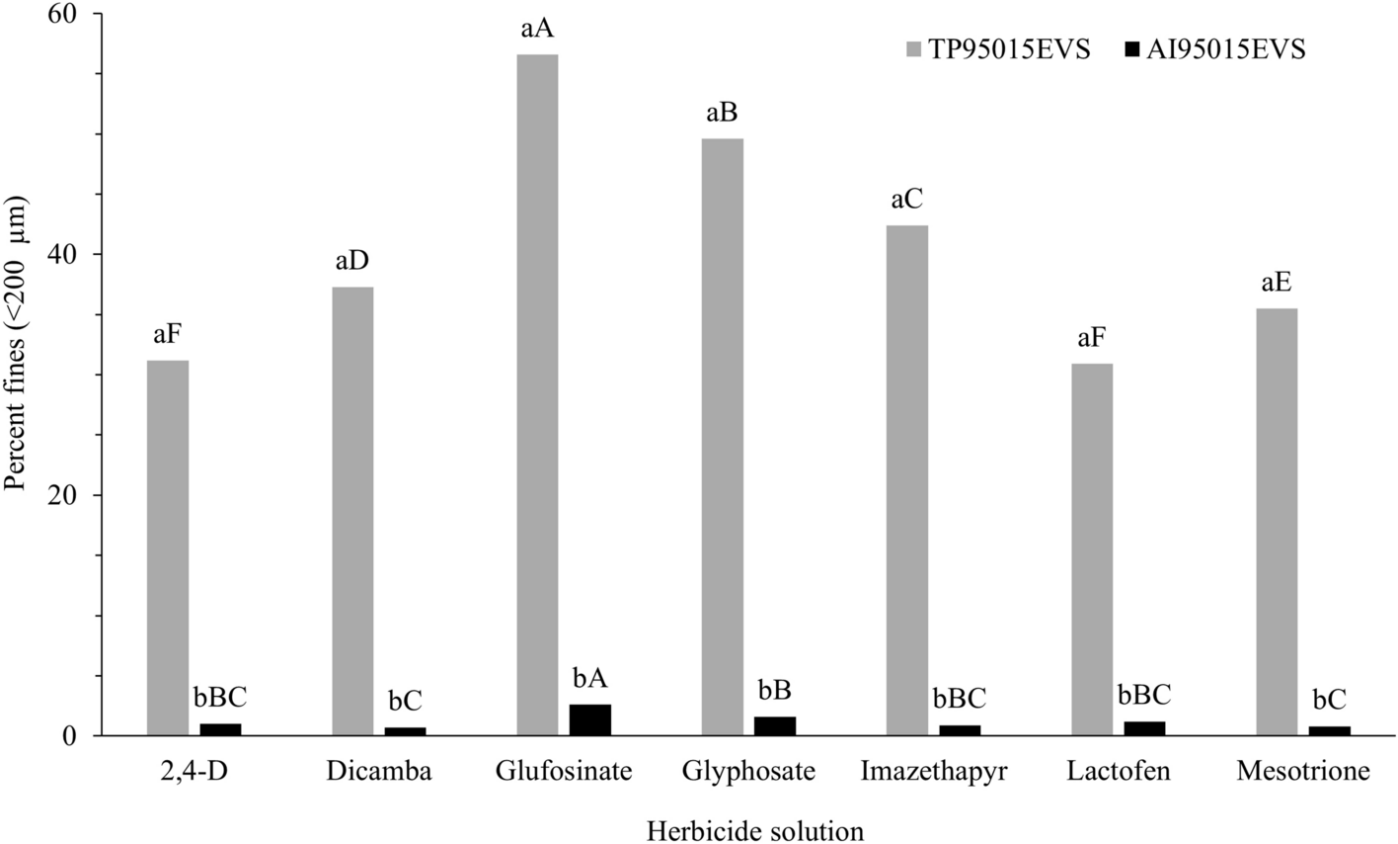
Percentage of droplets smaller than 200 μm produced by seven herbicide solutions sprayed with two different nozzle types. Differences between herbicide solutions within nozzle type are indicated by different uppercase letters, whereas differences between nozzles within each herbicide solution are indicated by lowercase letters (α = 0.05).

Nozzle design and herbicide solution influenced D_V0.1_, D_V0.5_, D_V0.9_, and the percent of driftable fines values (*P* < 0.0001). The TP nozzle had a smaller droplet diameter (Table 2) and a greater proportion of driftable fines than the AI nozzle across herbicides (Fig. 2). In general, the TP nozzle had a “fine” spray classification with the lowest D_V0.5_ values observed for glufosinate (182 µm) and glyphosate (201 µm) (Table 2). For the TP nozzle, the lowest drift potential was observed for 2,4-D and lactofen across herbicides. The application of herbicides with an AI nozzle resulted in the formation of larger droplets (“extremely coarse” and “ultra coarse” spray classifications), which, as a result, resulted in a substantial decrease in drift potential. Glufosinate had the highest potential for drift across all herbicide solutions tested for the TP nozzle (56.6%) and the AI nozzle (2.6%) (Fig. 2). Similarly, Creech et al.^24^ reported a decrease in Dv_0.5_ of about 18% for glufosinate compared to water-alone treatment pooled across nozzles. The differences in drift potential and droplet size categories demonstrate the influence of both nozzle type and the specific herbicide used, which may result in variations in spray deposition. The nozzle design emerged as the most critical determinant, in contrast to the composition of the herbicide solution, which exhibited a comparatively minimal effect, corroborating with previous research reports^24,25,52,54^.

**Table 2:** Droplet size distribution for TP95015EVS and AI95015EVS nozzles spraying herbicide solutions evaluated in the spray drift study.

Herbicide solution	TP95015EVS^a^							AI95015EVS^a^						
	D_V0.1_		D_V0.5_		D_V0.9_		SC^b^	D_V0.1_		D_V0.5_		D_V0.9_		SC^b^
	μm							μm						
2,4-D	129	bA	251	bA	394	bA	F	391	aC	740	aD	1089	aE	EC
Dicamba	114	bC	236	bC	396	bA	F	433	aB	836	aA	1262	aA	UC
Glufosinate	79	bF	182	bF	342	bC	F	335	aF	720	aE	1118	aD	EC
Glyphosate	88	bE	201	bE	354	bBC	F	385	aD	796	aC	1169	aC	EC
Imazethapyr	103	bD	220	bD	370	bAB	F	441	aA	845	aA	1254	aA	UC
Lactofen	132	bA	250	bAB	385	bA	F	375	aE	708	aF	1048	aF	EC
Mesotrione	120	bB	240	bBC	392	bA	F	437	aA	820	aB	1201	aB	UC

^a^D_V0.1_, D_V0.5_, and D_V0.9_ represent the droplet size such that 10, 50, and 90% of the spray volume is contained in droplets equal or lesser diameters, respectively. Lowercase letters compare nozzles within herbicide and uppercase letters compare herbicides within nozzle. Means by the same letter are not different (P ≤ 0.05).

^b^The spray classifications (SC) for this study were based on reference curves created from reference nozzle data at the Pesticide Application Technology Laboratory as described by ASABE S572.3 where F = Fine, EC = Extremely Coarse, and UC = Ultra Coarse.

### Spray deposition is dependent on nozzle type and herbicide

Pooling herbicides across nozzles indicates that TP and AI nozzles had 5% of the spray deposits reaching 5.9 and 2.0 m downwind, respectively. Similar results have been reported in the literature, where employment of nozzles with an air-inclusion port decreased drift potential^22,38,51^. Adding a pre-orifice and air-inclusion port allows pressure to drop within a nozzle and the introduction of air into the herbicide solution, directly increasing droplet size and decreasing overall drift potential^25,36^.

**Fig. 3:**
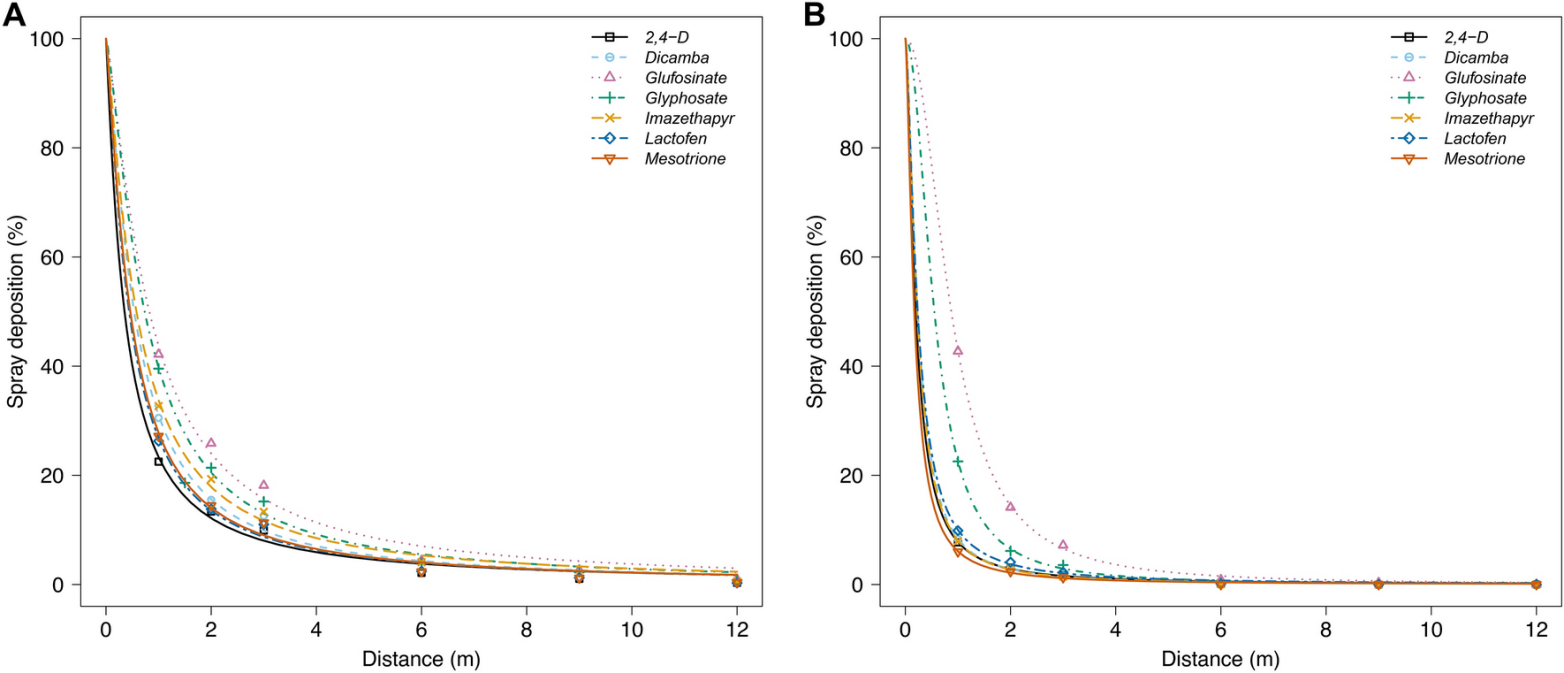
Spray deposition on mylar cards from herbicide drift in a low-speed wind tunnel using (**A**) TP95015EVS and (**B**) AI95015EVS nozzles (TeeJet Technologies Spraying Systems Co., Glendale Heights, IL, USA) at 230 kPa. Mean estimates were determined from eight replications across two experimental runs. Parameter estimates for the models are in Table 2.

Tracer spray deposition results for comparison made across nozzles show a higher drift profile for the TP nozzle (Fig. 3A) compared to the AI nozzle (Fig. 3B). To determine the inflection point, slope (*b*), and distance to 50% application spray deposition (*e*), log-logistic model was used with parameters presented in Table 3. A larger *e* value indicates that greater distances were required to observe 50% spray deposition, indicating more spray drift potential. Results indicate that the distance to 50% of application spray deposition (parameter *e*) decreased by at least 25% for most of the evaluated herbicides by changing the nozzle from TP to AI. There was no difference between the distances where 50% spray deposition was estimated for glufosinate herbicide, even when the nozzle type was changed. A similar trend was observed in the droplet size distribution study with the lowest D_V0.5_ values (Table 2) followed by the most significant proportion of percent of fines < 200 µm (Fig. 2) for glufosinate. Those findings may be partially attributed to the interaction of nozzle type with herbicide formulation^24,58–60^. As previously reported, product formulation types could affect droplet size and drift reduction by changes in physical properties (including surface tension and viscosity of sprayed solutions) that directly influence atomization through specific nozzle types^60,61^. Therefore, identifying products is especially important when reproducing or extending previous research findings, as relying solely on active ingredients may not provide enough detail to replicate experimental conditions accurately.

**Table 3:** Log-logistic model parameters and standard errors for TP95015EVS and AI95015EVS nozzles spraying herbicide solutions evaluated in the spray deposition study.

Herbicide solution	Log-logistic model parameters^a^			
	TP95015EVS		AI95015EVS	
	b	e	b	e
		m		m
2,4-D	1.14 ± 0.08	0.35 ± 0.04	1.53 ± 0.19	0.20 ± 0.04
Dicamba	1.28 ± 0.07	0.54 ± 0.03	1.66 ± 0.20	0.24 ± 0.04
Glufosinate	1.30 ± 0.05	0.82 ± 0.03	2.16 ± 0.05	0.87 ± 0.01
Glyphosate	1.35 ± 0.06	0.73 ± 0.03	2.07 ± 0.10	0.55 ± 0.02
Imazethapyr	1.23 ± 0.06	0.57 ± 0.03	1.63 ± 0.20	0.22 ± 0.04
Lactofen	1.22 ± 0.08	0.44 ± 0.04	1.51 ± 0.15	0.23 ± 0.04
Mesotrione	1.23 ± 0.07	0.46 ± 0.04	1.55 ± 0.25	0.17 ± 0.05

^a^b parameter corresponds to the slope at the inflection point; e parameter corresponds to the distance estimated for 50% of spray deposition.

### Herbicide downwind drift affected industrial hemp biomass

**Fig. 4:**
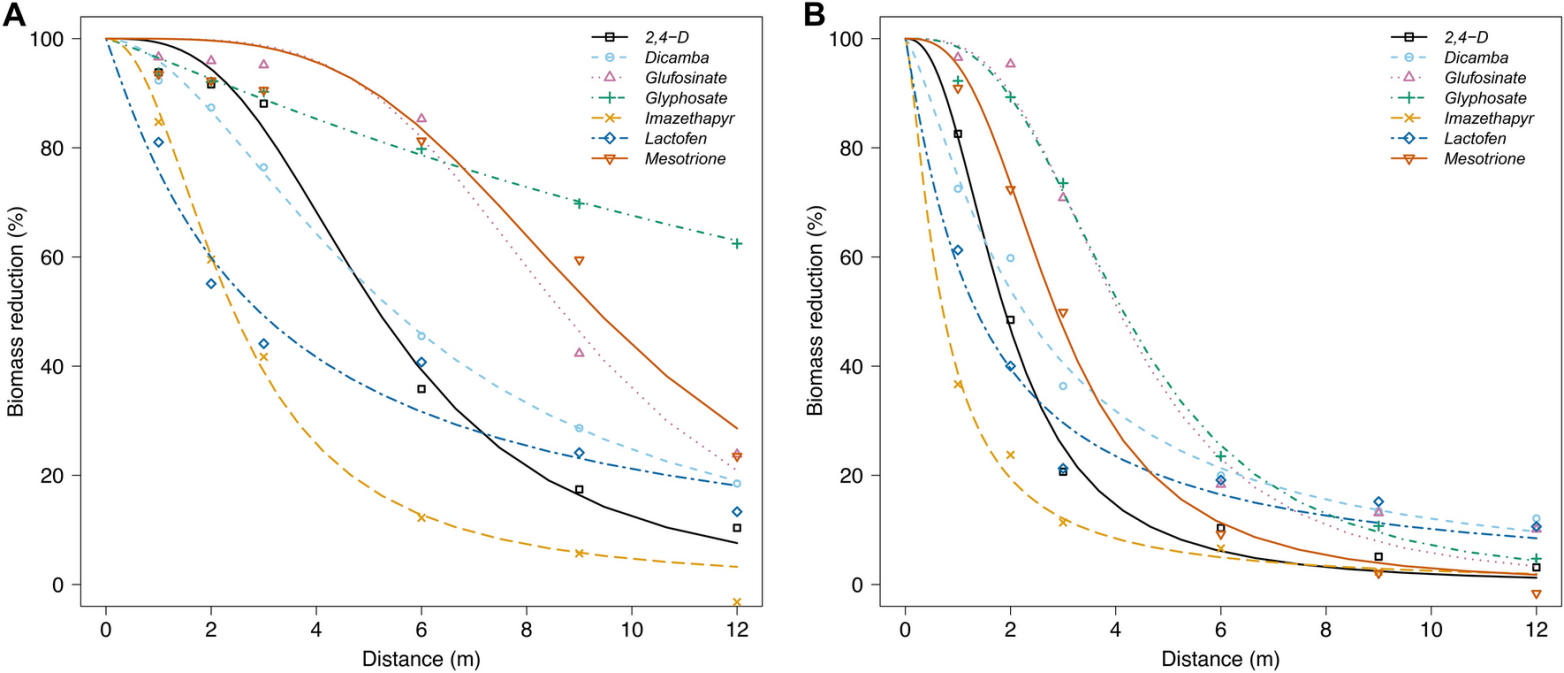
Industrial hemp biomass reduction caused by herbicide drift in a low-speed wind tunnel from applications with (**A**) TP95015EVS and (**B**) AI95015EVS nozzles (TeeJet Technologies Spraying Systems Co., Glendale Heights, IL, USA) at 230 kPa. Mean estimates were determined from eight replications across two experimental runs. Parameter estimates for the models are in Table 3.

The susceptibility to biomass reduction greatly depended on nozzle type and herbicide solution. For TP (Fig. 4A) and AI (Fig. 4B), the nozzles used for herbicide drift simulation results indicate that as downwind distance increased, there was less impact on overall industrial hemp biomass as observed for spray deposition.

Log-logistic model parameter estimates indicate the greatest susceptibility of industrial hemp to glyphosate, mesotrione, and glufosinate (Table 4). Changing the nozzle from TP to AI reduced the distance in meters at which 50% biomass reduction occurred from 19.31 (± 4.32) to 4.14 (± 0.21) for glyphosate. Because the furthest distance evaluated in the study was 12 m due to limitations of wind tunnel length, the distance for 50% biomass reduction for glyphosate was extrapolated based on the data points collected. Sensitivity to glyphosate is due to interference with the shikimic acid production pathway by inhibiting 5-enolpyruvylshikimate-3-phosphate synthase^62^ necessary for the normal development of plants and synthesis of aromatic substances like lignin^63–65^. Industrial hemp susceptibility to glyphosate has been previously documented, even when applied at sub-labeled rates^41,66,67^. Ortmeier-Clarke et al.^41^ were unable to fit a dose–response model for industrial hemp due to high susceptibility associated with POST application of glyphosate across a range of doses (157.5 to 1260 g ai ha^−1^). The susceptibility to glyphosate has been documented in other commodity crops, including wheat^15^, corn (non-glyphosate-tolerant)^68,69^, and rice^70^. In these crops, final biomass reduction depended on the selected hybrid or cultivar and the growth stage at exposure time. Given the limited information available on hemp in the literature, the impact of glyphosate on different hemp cultivars and growth stages remains uncertain. Therefore, the results of this study should be interpreted with caution and may vary depending on the hemp cultivars and growth stages used. Reddy et al.^69^ findings associated with field drift from an aerial application of glyphosate on non-glyphosate-tolerant corn shows that at an airspeed of 3.11 m s^−1^, the distance for 50% of shoot dry weight reduction was estimated to be at 19.42 (± 0.46) m from the sprayed area three weeks after application. Following similar susceptibility observed with other commodities, hemp deserves special care in the predominant corn and soybean cropping systems where glyphosate applications are still dominant^71^.

**Table 4:** Log-logistic model parameters and standard errors for TP95015EVS and AI95015EVS nozzles spraying herbicide solutions evaluated in the biomass reduction study.

Herbicide solution	Log-logistic model parameters^a^			
	TP95015EVS		AI95015EVS	
	b	e	b	e
		m		m
2,4-D	2.97 ± 0.36	5.18 ± 0.25	2.37 ± 0.34	1.89 ± 0.11
Dicamba	1.86 ± 0.20	5.49 ± 0.32	1.33 ± 0.15	2.25 ± 0.18
Glufosinate	4.10 ± 0.60	8.67 ± 0.28	3.11 ± 0.39	4.06 ± 0.21
Glyphosate	1.12 ± 0.25	19.31 ± 4.31	2.91 ± 0.34	4.14 ± 0.21
Imazethapyr	2.13 ± 0.24	2.43 ± 0.14	1.39 ± 0.25	0.72 ± 0.12
Lactofen	1.06 ± 0.11	2.91 ± 0.25	1.09 ± 0.16	1.35 ± 0.16
Mesotrione	3.66 ± 0.68	9.34 ± 0.33	2.81 ± 0.36	2.87 ± 0.14

^a^b parameter corresponds to the slope at the inflection point; e parameter corresponds to the distance estimated for 50% industrial hemp biomass reduction.

Switching from TP to AI nozzles significantly reduced the distance at which 50% biomass reduction occurred for mesotrione and glufosinate, underscoring the importance of nozzle selection in herbicide application. For mesotrione and glufosinate, changing the nozzle from TP to AI reduced the distance at which 50% biomass reduction occurred by about 3.3- and 2.1-fold, respectively. Despite the significant decrease in the estimated 50% biomass reduction distance using the AI nozzle, industrial hemp exhibited higher sensitivity levels than other tested herbicides, particularly mesotrione and glufosinate. Ortmeier-Clarke et al.^41^ found that high sensitivity to mesotrione and glufosinate resulted in a 10% biomass reduction at effective doses of 0.005 (± 0.03) and 21.0 (± 10.1) g ai ha^−1^, respectively, when applied POST. This corroborates that hemp is highly susceptible to mesotrione and glufosinate, as low doses can greatly reduce biomass accumulation. Consequently, selecting appropriate application parameters is crucial to prevent a negative impact on hemp growth and development. It is also essential to highlight the anticipated introduction of the next generation of genetically modified corn and soybean traits, currently known as the “HT5 trait”. This trait encompasses tolerance to a range of herbicides, including glyphosate, glufosinate, dicamba, 2,4-D, glufosinate, 4-Hydroxyphenylpyruvate dioxygenase (HPPD Inhibitors; WSSA Group 27), and Protoporphyrinogen Oxidase Inhibitors (PPO) inhibitors^72^. The emergence of these traits will likely lead to an intensified use of listed herbicide traits, which is necessary to consider due to the observed sensitivity of hemp. Such an increase poses a significant risk of herbicide drift, potentially affecting susceptible crops, including hemp.

Results indicate that industrial hemp was more susceptible to dicamba compared to 2,4-D. According to the inflection point-slope (parameter *b*), for both nozzles tested, there is a faster curve decay for 2,4-D than for dicamba (Table 4). Even though a multi-purpose variety of hemp was used (CRS-1 for food, fiber, and CBD; Hemp Genetics International, Saskatoon, SK, Canada), the findings from current study do not corroborate with Ortmeier-Clarke et al.^41^, who found the effective dose for 50% biomass reduction to be at 79.7 (± 6.8) and 122.6 (± 11.0) g ae ha^−1^ for 2,4-D and dicamba, respectively. The discrepancy in the results may be genetic variability, as previously mentioned for glyphosate, due to selective or artificial breeding selection methods to meet specific environmental needs. In addition, most previous studies assessed industrial hemp susceptibility to direct herbicide application over the top of the plants instead of indirect exposure to herbicide drift^41,50^.

Regardless of the nozzle used, imazethapyr and lactofen resulted in lower hemp biomass reduction levels when compared to the other herbicides tested. Even under high drift scenarios with the TP nozzle, the estimated 50% biomass reduction distances for imazethapyr and lactofen were no greater than 2.43 to 2.91 m downwind, respectively. The observed findings for these two active ingredients do not consistently align with published literature. For example, Flessner et al.^50^ reported no impact on height when imazethapyr (200 g ai ha^−1^) was applied POST to 25–30 cm tall industrial hemp plants. Still, there was a 51% biomass reduction relative to the non-treated control. The differing application rates between Flessner et al.^50^ and the current study (200 versus 70 g ai ha^−1^) might explain the varied responses. Similarly, hemp tolerance varied for other PPO inhibitors. Ortmeier-Clarke et al. (2022) observed biomass reduction above 75% and 35–65% for two hemp cultivars when lactofen was applied POST at 220 and 27.5 g ai ha^−1^, respectively. High sensitivity (approximately 70% biomass reduction) to direct application of fomesafen and acifluorfen has been reported in the literature^41,50^. These findings underscore the complexity and variability in hemp response to various PPO-inhibiting herbicides, emphasizing the necessity for ongoing research. As agricultural practices and herbicide technologies progress, including the development of the HT5 corn and soybean traits, there is an increasing need to adapt and respond to these changes in a well-informed manner^72^.

The present study examines the effects of systemic and contact herbicides on hemp. As previously reported, systemic herbicides, including glyphosate, mesotrione, 2,4-D, and dicamba, are effective at lowering spray carrier volumes and coverage^73,74^. However, their systemic nature presents a distinct risk in drift scenarios, as even low doses can be absorbed and translocated within plants, potentially causing a greater impact on non-target species like hemp. Increased activity on cotton with low doses of 2,4-D and dicamba was observed in lower spray carrier volumes with more concentrated droplets^75^. Previous studies conducted on herbicide efficacy have shown that responses are influenced by carrier volume^73,76^. Nonetheless, available research on industrial hemp susceptibility to herbicides, including the studies by Flessner et al.^50^ and Ortmeier-Clarke et al.^41^, has primarily focused on sub-labeled doses applied directly over the plants. However, even at similar dose ranges, the impact of actual herbicide drift might differ significantly from those of these controlled applications. Wang and Liu^77^ emphasized that the concentration of active ingredients within droplets might influence the diffusion process during foliar uptake. Oppositely, for contact herbicides examined, glufosinate, lactofen, and imazethapyr typically require higher carrier volumes, with greater coverage crucial for effectiveness. The drift implications for these herbicides differ; reduced coverage may reduce their impact^24,73^. As a result, non-systemic activity indicates that they are less likely to impact non-target plants significantly. The findings underscore the importance of considering herbicide activity and application methods, particularly in potential drift, to minimize unintended environmental consequences.

Besides employing additional drift-mitigation techniques (i.e., nozzle, adjuvants, or others), current and future commercial and private pesticide applicators should follow the labeled application recommendations and buffer zone requirements of 73.2 m and 9.2 m for dicamba^78^ and 2,4-D^79^, respectively. Determining appropriate buffer widths for herbicides lacking specified buffer zones depends on risk assessment and drift potential related to the chosen pesticide application parameters. Unless otherwise specified on the product label, it is recommended that industrial hemp fields maintain no-spray buffer zones of at least 15 m (i.e., the standard buffer width for non-organic fields, potentially representing a worst-case scenario) to protect surrounding vegetation when employing herbicides without designated buffer zones^80^. However, no-spray buffer zones may increase based on risk assessment after site inspection to avoid unintended area exposure to prohibited active ingredients even when drift-mitigating nozzles are used^80^. Additional tactics that can help with the buffer zone width changes may include additional no-spray barriers consisting of row crops such as corn^52^, hedgerows^81^, and the employment of hooded sprayers to mitigate drift potential^82,83^. As a result, the practices mentioned above aim to avoid unintended off-target movement and adverse effects on industrial hemp growth and development.

In conclusion, this study highlights the critical need for herbicide drift mitigation techniques in industrial hemp production to prevent adverse effects on biomass production and ensure high-quantity and quality plant materials for processing. This research provides valuable insights into the impact of nozzle design and herbicide solution on droplet size distribution, driftable fines, spray deposition, and industrial hemp biomass accumulation. Notably, nozzle type and herbicide solution significantly influence droplet size distribution, drift potential, and herbicide damage to industrial hemp. Substituting TP nozzles with AI can reduce spray drift and prevent negative impacts on industrial hemp biomass reduction, particularly when exposed to glyphosate, mesotrione, and glufosinate drift. Imazethapyr and lactofen spray drift resulted in lower hemp biomass reduction levels when compared to the other herbicides tested in this study. These findings have practical implications for agricultural pesticide applicators to make informed decisions when selecting nozzle types and herbicide solutions to minimize off-target movement and avoid potential crop damage. If additional drift-reduction techniques (drift-reducing adjuvants or hooded sprayers) or buffer zones are not employed, overall plant biomass (presumably yield of flowers, fiber, and grain) might be reduced. Ongoing research is necessary to develop effective practices for agricultural pest management, including the implementation of drift-reduction techniques and no-spray buffer zones when hemp is planted in adjacent fields. Additionally, future studies should examine the impact of herbicide drift on industrial hemp during the reproductive stage and potential changes in tetrahydrocannabinol, other cannabinoids, and herbicide residue accumulation in plants. These findings can help promote sustainable agricultural practices that balance effective pest management with environmental stewardship and economic viability.

## Declarations

### Data availability

The datasets generated and/or analyzed during the current study are available from the corresponding author on a reasonable request.

## References

[1] Adesina, I., Bhowmik, A., Sharma, H. & Shahbazi, A. A Review on the current state of knowledge of growing conditions, agronomic soil health practices and utilities of hemp in the United States. *Agriculture* **10**, 129 (2020).

[2] Amaducci, S. et al. Key cultivation techniques for hemp in Europe and China. *Ind. Crops Prod.* **68**, 2–16 (2015).

[3] Mark, T. *et al.* Economic Viability of Industrial Hemp in the United States: A Review of State Pilot Programs. *U.S. Dep. of Agric., Econ. Res. Serv.* https://www.ers.usda.gov/webdocs/publications/95930/eib-217.pdf?v=9065.7 (2020).

[4] Karus, M. & Vogt, D. European hemp industry: Cultivation, processing and product lines. *Euphytica* **140**, 7–12 (2004).

[5] Olson, D. W., Thornsbury, S. D. & Scott, S. Hope for Hemp: New Opportunities and Challenges for an Old Crop. *U.S. Dep. of Agric., Econ. Res. Serv.* https://www.ers.usda.gov/amber-waves/2020/june/hope-for-hemp-new-opportunities-and-challenges-for-an-old-crop/ (2020).

[6] Johnson, R. Hemp as an Agricultural Commodity. *Congressional Res. Serv.* https://sgp.fas.org/crs/misc/RL32725.pdf (2018).

[7] Anonymous. National Hemp Report (February 2022). *U.S. Dep. of Agric., National Agric. Stat. Serv.* https://release.nass.usda.gov/reports/hempan22.pdf (2022).

[8] Dodson, L. Recent Trends in GE Adoption. *U.S. Dep. of Agric., Econ. Res. Serv.* https://www.ers.usda.gov/data-products/adoption-of-genetically-engineered-crops-in-the-us/recent-trends-in-ge-adoption/ (2020).

[9] Harker, K. N. & O’Donovan, J. T. Recent weed control, weed management, and integrated weed management. *Weed Technol.* **27**, 1–11 (2013).

[10] Kniss, A. R. Genetically engineered herbicide-resistant crops and herbicide-resistant weed evolution in the United States. *Weed Sci.* **66**, 260–273 (2018).

[11] Reddy, K. N. Glyphosate-resistant soybean as a weed management tool: Opportunities and challenges. *Weed Biol. Manag.* **1**, 193–202 (2001).

[12] Al-Khatib, K. & Peterson, D. Soybean (Glycine max) response to simulated drift from selected sulfonylurea herbicides, dicamba, glyphosate, and glufosinate. *Weed Technol.* **13**, 264–270 (1999).

[13] Alves, G. S. et al. Spray drift and efficacy from glyphosate and 2,4-D applications with adjuvants. *Biosci. J.* **36**, 876–885 (2020).

[14] Jones, G. T., Norsworthy, J. K. & Barber, T. L. Off-target movement of diglycolamine dicamba to non-dicamba soybean using practices to minimize primary drift. *Weed Technol.* **33**, 24–40 (2019).

[15] Roider, C. A., Griffin, J. L., Harrison, S. A. & Jones, C. A. Wheat response to simulated glyphosate drift. *Weed Technol.* **21**, 1010–1015 (2007).

[16] Soltani, N. et al. Off-target movement assessment of dicamba in North America. *Weed Technol.* **34**, 318–330 (2020).

[17] Werle, R. et al. Survey of Nebraska farmers’ adoption of dicamba-resistant soybean technology and dicamba off-target movement. *Weed Technol.* **32**, 754–761 (2018).

[18] Briand, O., Bertrand, F., Seux, R. & Millet, M. Comparison of different sampling techniques for the evaluation of pesticide spray drift in apple orchards. *Sci. Total Environ.* **288**, 199–213 (2002). 11991524 10.1016/s0048-9697(01)00961-5

[19] Matthews, G., Bateman, R. & Miller, P. *Pesticide Application Methods*, 4th edn. (Wiley-Blackwell, 2014).

[20] Pivato, A. et al. An integrated model-based approach to the risk assessment of pesticide drift from vineyards. *Atmos. Environ.* **111**, 136–150 (2015).

[21] Van der Werf, H. M. G. Assessing the impact of pesticides on the environment. *Agric. Ecosyst. Environ.* **60**, 81–96 (1996).

[22] Alves, G. S. et al. Dicamba spray drift as influenced by wind speed and nozzle type. *Weed Technol.* **31**, 724–731 (2017).

[23] Hewitt, A. J. Spray drift: impact of requirements to protect the environment. *Crop Prot.* **19**, 623–627 (2000).

[24] Creech, C. F., Henry, R. S., Fritz, B. K. & Kruger, G. R. Influence of herbicide active ingredient, nozzle type, orifice size, spray pressure, and carrier volume rate on spray droplet size characteristics. *Weed Technol.* **29**, 298–310 (2015).

[25] Dorr, G. et al. A comparison of initial spray characteristics produced by agricultural nozzles. *Crop Prot.* **53**, 109–117 (2013).

[26] Havens, P. L. et al. Field measurements of drift of conventional and drift control formulations of 2,4-d plus glyphosate. *Weed Technol.* **32**, 550–556 (2018).

[27] Nordby, A. & Skuterud, R. The effects of boom height, working pressure and wind speed on spray drift. *Weed Res.* **14**, 385–395 (1974).

[28] Rodrigues, A. O. *et al.* Influence of nozzle type, speed, and pressure on droplet size and weed control from glyphosate, dicamba, and glyphosate plus dicamba. *ASTM Intern.* https://www.astm.org/stp161020170249.html (2016).

[29] Vieira, B. C. et al. Particle drift potential of glyphosate plus 2,4-D choline pre-mixture formulation in a low-speed wind tunnel. *Weed Technol.* **34**, 520–527 (2020).

[30] Nuyttens, D., Baetens, K., Schampheleire, M. D. & Sonck, B. Effect of nozzle type, size and pressure on spray droplet characteristics. *Biosyst. Eng.* **3**, 333–345 (2007).

[31] Nuyttens, D. *et al*. Drift-reducing nozzles and their biological efficacy. *Commun. Agric. Appl. Biol. Sci.* **74**, 47–55 (2009). 20218510

[32] Ramsdale, B. K. & Messersmith, C. G. Drift-reducing nozzle effects on herbicide performance. *Weed Technol.* **15**, 453–460 (2001).

[33] Souza, L. L. & Moretti, M. L. Chemical control of suckers in hazelnut orchards of western Oregon. *Weed Technol.* **34**, 863–869 (2020).

[34] Yates, W. E., Cowden, R. E. & Akesson, N. A. Drop size spectra from nozzles in high-speed airstreams. *Trans. ASAE* **28**, 405–410 (1985).

[35] Perine, J., Anderson, J. C., Kruger, G. R., Abi-Akar, F. & Overmyer, J. Effect of nozzle selection on deposition of thiamethoxam in Actara^®^ spray drift and implications for off-field risk assessment. *Sci. Total Environ.* **772**, 144808 (2021). 33770886 10.1016/j.scitotenv.2020.144808

[36] Derksen, R. C., Ozkan, H. E., Fox, R. D. & Brazee, R. D. Droplet spectra and wind tunnel evaluation of venturi and pre-orifice nozzles. *Trans. ASAE* **42**, 1573–1580 (1999).

[37] Guler, H. et al. Spray characteristics and drift reduction potential with air induction and conventional flat-fan nozzles. *Trans. ASABE* **50**, 745–754 (2007).

[38] Vieira, B. C. *et al*. Response of Amaranthus spp. following exposure to sublethal herbicide rates via spray particle drift. *PLoS One* **14**, e0220014 (2019). 10.1371/journal.pone.0220014 PMC6638980 31318947

[39] Anonymous. Pesticide products registered for use on hemp, pesticide registration. *U.S. Environ. Prot. Agency* https://www.epa.gov/%20pesticide-registration/pesticide-products-registered-use-hemp (2022).

[40] Tsaliki, E. *et al*. Fibre and seed productivity of industrial hemp (Cannabis sativa L.) varieties under mediterranean conditions. *Agronomy* **11**, 171 (2021).

[41] Ortmeier-Clarke, H. J., Oliveira, M. C., Arneson, N. J., Conley, S. P. & Werle, R. Dose response screening of industrial hemp to herbicides commonly used in corn and soybean. *Weed Technol.* **36**, 245–252 (2022).

[42] Michlig, N., Lehotay, S. J., Lightfield, A. R., Beldoménico, H. & Repetti, M. R. Validation of a high-throughput method for analysis of pesticide residues in hemp and hemp products. *J. Chrom.* **1645**, 462097 (2021). 10.1016/j.chroma.2021.462097 33848664

[43] Seltenrich, N. Into the weeds: regulating pesticides in cannabis. *Environ. Health Perspect.*10.1289/EHP5265 (2019). 10.1289/EHP5265 PMC6785225 31021196

[44] Bales, S. R. & Sprague, C. L. Sensitivity of dry edible bean to dicamba and 2,4-D. *Weed Technol.* **34**, 117–124 (2020).

[45] Buol, J. T. et al. Effect of growth stage on cotton response to a sublethal concentration of dicamba. *Weed Technol.* **33**, 1–8 (2019).

[46] Calzolari, D. et al. High added-value compounds from Cannabis threshing residues. *Ind. Crops Prod.* **108**, 558–563 (2017).

[47] Dintelmann, B. R., Warmund, M. R., Bish, M. D. & Bradley, K. W. Investigations of the sensitivity of ornamental, fruit, and nut plant species to driftable rates of 2,4-D and dicamba. *Weed Technol.* **34**, 331–341 (2019).

[48] Johnson, V. A. et al. Cotton, peanut, and soybean response to sublethal rates of dicamba, glufosinate, and 2,4-D. *Weed Technol.* **26**, 195–206 (2012).

[49] Andre, C. M., Hausman, J. F. & Guerriero, G. Cannabis sativa: the plant of the thousand and one molecules. *Front. Plant Sci.* **7** (2016). 10.3389/fpls.2016.00019 PMC4740396 26870049

[50] Flessner, M. L., Bryd, J., Bamber, K. W. & Fike, J. H. Evaluating herbicide tolerance of industrial hemp (Cannabis sativa L.). *Crop Sci.* **60**, 419–427 (2020).

[51] Creech, C. F., Moraes, J. G., Henry, R. S., Luck, J. D. & Kruger, G. R. The impact of spray droplet size on the efficacy of 2,4-d, atrazine, chlorimuron-methyl, dicamba, glufosinate, and saflufenacil. *Weed Technol.* **30**, 573–586 (2016).

[52] Vieira, B. C. *et al*. Spray particle drift mitigation using field corn (*Zea**mays* L.) as a drift barrier. *Pest Manag. Sci.* **74**, 2038–2046 (2018). 10.1002/ps.5041 29688591

[53] Anonymous. Spray nozzle classification by droplet spectra. *A. Soc. Agric. Biol. Eng.* https://elibrary.asabe.org/abstract.asp?aid=51101&t=3&redir=&redirType= (2020).

[54] Brankov, M. *et al*. Particle drift simulation from mesotrione and rimsulfuron plus thifensulfuron-methyl mixture through two nozzle types to field and vegetable crops. *Environ. Sci. Pollut. Res.* **30** (2023). 10.1007/s11356-022-24938-x 36580245

[55] Oliveira, M. C. *et al*. Additive design: the concept and data analysis. *Weed Res.* **58** (2018).

[56] Knezevic, S. Z., Streibig, J. C. & Ritz, C. Utilizing R software package for dose-response studies: the concept and data analysis. *Weed Technol.* **21**, 840–848 (2007).

[57] Ritz, C., Baty, F., Streibig, J. C. & Gerhard, D. Dose-response analysis using R. *PLoS One* **10**, e0146021 (2015). 26717316 10.1371/journal.pone.0146021 PMC4696819

[58] Vieira, B. C. et al. Spray drift potential of dicamba plus S-metolachlor formulations. *Pest Manag. Sci.* **78**, 1538–1546 (2022). 34964546 10.1002/ps.6772

[59] Meyer, C. J., Norsworthy, J. K., Kruger, G. R. & Barber, T. L. Effect of nozzle selection and spray volume on droplet size and efficacy of engenia tank-mix combinations. *Weed Technol.* **30**, 377–390 (2016).

[60] Mueller, T. C. & Womac, A. R. Effect of formulation and nozzle type on droplet size with isopropylamine and trimesium salts of glyphosate. *Weed Technol.* **11**, 639–643 (1997).

[61] Hilz, E. & Vermeer, A. W. P. Spray drift review: The extent to which a formulation can contribute to spray drift reduction. *Crop Prot.* **44**, 75–83 (2013).

[62] Ghosh, S., Chisti, Y. & Banerjee, U. C. Production of shikimic acid. *Biotechnol. Adv.* **30**, 1425–1431 (2012). 22445787 10.1016/j.biotechadv.2012.03.001

[63] Gandolfi, S., Ottolina, G., Riva, S., Fantoni, G. P. & Patel, I. Complete chemical analysis of carmagnola hemp hurds and structural features of its components. *BioResources* **8**, 2641–2656 (2013).

[64] Liu, Q., Luo, L. & Zheng, L. Lignins: biosynthesis and biological functions in plants. *Int. J. Mol. Sci.* **19** (2018). 10.3390/ijms19020335 PMC5855557 29364145

[65] Stevulova, N. et al. Properties characterization of chemically modified hemp hurds. *Materials* **7**, 8131–8150 (2014). 28788294 10.3390/ma7128131 PMC5456447

[66] Horowitz, M. Herbicidal treatments for control of Cannabis sativa L. *Bull. Narcot.* **29**, 75–84 (1977). 585583

[67] Sosnoskie, L. M. & Maloney, E. Industrial hemp sensitivity to pre- and postemergence herbicides. *Weed Sci. Soc. of America*. https://wssa.net/wp-content/uploads/WSSA-Virtual-Annual-Meeting-all-abstracts-updated-4-12-2021ab_FINAL.pdf (2021).

[68] Barnes, E. R. et al. Dose response of yellow and white popcorn hybrids to glyphosate, a premix of 2,4-D choline and glyphosate, or dicamba. *Agron. J.* **112**, 2956–2967 (2020).

[69] Reddy, K. N. *et al*. Biological responses to glyphosate drift from aerial application in non-glyphosate-resistant corn. *Pest Manag. Sci.* **66**, 1148–1154 (2010). 10.1002/ps.1996 20662010

[70] Koger, C. H. et al. Rice (*Oryza**sativa*) response to drift rates of glyphosate. *Pest Manag. Sci.* **61**, 1161–1167 (2005). 16189844 10.1002/ps.1113

[71] Fernandez-Cornejo, J. Glyphosate use is more widespread in soybean than in corn production. *U.S. Dep. of Agric. Econ. Res. Serv.* https://www.ers.usda.gov/data-products/chart-gallery/gallery/chart-detail/?chartId=78187 (2015).

[72] Reither, B. R&D pipeline update: The beginning of what’s next. *Bayer* https://www.bayer.com/sites/default/files/BayerCMD2021_CropScience_RandD_Presentation.pdf (2021).

[73] Butts, T. R. et al. Spray droplet size and carrier volume effect on dicamba and glufosinate efficacy. *Pest Manag. Sci.* **74**, 2020–2029 (2018). 10.1002/ps.4913 29536620

[74] Vranjes, F., Vrbnicanin, S. Nedeljkovic, D. Savic, A. & Bozic, D. The response of *Chenopodium**album* L. and *Abutilon**theophrasti* Medik. to reduced doses of mesotrione. *J. Environ. Sci. Health* **54**, 615–621 (2019). 10.1080/03601234.2019.1616980 31116075

[75] Smith, H. C., Ferrell, J. A., Webster, T. M. & Fernandez, J. V. Cotton response to simulated auxin herbicide drift using standard and ultra-low carrier volumes. *Weed Technol.* **31**, 1–9 (2017).

[76] Creech, C. F. et al. Performance of postemergence herbicides applied at different carrier volume rates. *Weed Technol.* **29**, 611–624 (2015).

[77] Wang, C. J. & Liu, Z. Q. Foliar uptake of pesticides—present status and future challenge. *Pest. Biochem. Physiol.* **87**, 1–8 (2007).

[78] Anonymous. XtendiMax^®^ Herbicide with VaporGrip^®^ Technology. *Bayer CropScience* LP https://www.xtendimaxapplicationrequirements.com/pdf/xtendimax_label.pdf (2022).

[79] Anonymous. Enlist One^®^ with COLEX·D^®^ Technology. *Corteva Agriscience* LLC https://s3-us-west-1.amazonaws.com/agrian-cg-fs1-production/pdfs/Enlist_One_Label1i.pdf (2022).

[80] Anonymous. Buffer Zones—What are buffer zones and why does my farm need them? *U.S. Dep. of Agric., Agric. Mark. Serv.* https://www.ams.usda.gov/sites/default/files/media/6%20Buffer%20Zones%20FINAL%20RGK%20V2.pdf (2022).

[81] Lazzaro, L., Otto, S. & Zanin, G. Role of hedgerows in intercepting spray drift: Evaluation and modelling of the effects. *Agric., Ecosyst. Environ.* **123**, 317–327 (2008).

[82] Foster, H. C., Sperry, B. P., Reynolds, D. B., Kruger, G. R. & Claussen, S. Reducing herbicide particle drift: effect of hooded sprayer and spray quality. *Weed Technol.* **32**, 714–721 (2018).

[83] Vieira, B. C. et al. Hooded broadcast sprayer for particle drift reduction. *Pest Manag. Sci.* **78**, 1519–1528 (2021). 10.1002/ps.6770 34964248

